# Green Biosynthesized Silver Nanoparticles With Aqueous Extracts of *Ginkgo Biloba* Induce Apoptosis *via* Mitochondrial Pathway in Cervical Cancer Cells

**DOI:** 10.3389/fonc.2020.575415

**Published:** 2020-10-20

**Authors:** Zhen Xu, Qi Feng, Min Wang, Huange Zhao, Yingying Lin, Songlin Zhou

**Affiliations:** ^1^ Key Laboratory of Tropical Translational Medicine of the Ministry of Education and Hainan Provincial Key Laboratory of Tropical Medicine, Hainan Medical University, Haikou, China; ^2^ Jiangsu Provincial Key Laboratory of Veterinary Bio-pharmaceutical High-tech Research, Jiangsu Agri-Animal Husbandry Vocational College, Taizhou, China; ^3^ Health and Family Planning Commission of Wanzai County of Jiangxi Province, Yichun, China

**Keywords:** silver nanoparticles, *Ginkgo biloba*, apoptosis, mitochondrial signaling pathway, cervical cancer

## Abstract

Biosynthetic silver nanoparticles (AgNPs), specifically formed using medicinal plant extracts, have recently exhibited a remarkable therapeutic effect due to their anticancer potential. Here, we synthesized AgNPs using an aqueous extract of *Ginkgo bilob*a leaves and evaluated its activity against cervical cancer (CCa) and the related molecular mechanisms. The physiochemical properties of the AgNPs were measured by ultraviolet-visible spectrophotometry, nanometre particle size analyzer and transmission electron microscopy. The AgNPs effects on cell proliferation and apoptosis were investigated through MTT, MTS, and colony formation assay; Hoechst 33258 staining; and flow cytometry. The intracellular ROS and oxidative stress levels were assessed using the appropriate commercial kits. Apoptosis-related protein levels were determined by western blotting. We prepared a series of different sized ginkgo extract synthesized AgNPs (GB-AgNPs), and the smallest mean particle size was 40.2 ± 1.2 nm with low polydispersity (0.091 ± 0.011), zeta potential values showed -34.56 mV. Compared to the controls, the GB-AgNP treatment inhibited the cell proliferation and induced the apoptosis of HeLa and SiHa cells. In addition, GB-AgNP treatment led to markedly increased levels of intracellular ROS, the release of cytochrome c (Cyt C) from mitochondria into the cytosol and the cleavage of caspase -9 and -3 in both CCa cell lines. Moreover, NAC, an ROS scavenger, eliminated the effect of GB-AgNPs on the HeLa and SiHa cells. This study reveals that GB-AgNPs suppresses cancer cell proliferation and induces apoptosis by upregulating intracellular ROS generation and inducing the activation of the caspase-dependent mitochondrial apoptotic pathway in CCa cells. Thus, GB-AgNPs may be a potential alternative drug for CCa therapy.

## Introduction

In the past several decades, cancer has ranked as the second leading cause of death in worldwide (1). For females, cervical cancer (CCa) is becoming more prevalent, in terms of both incidence and mortality, which are ranked fourth, globally (2). Despite new chemotherapeutic agents, human papillomavirus vaccination and other therapeutic approaches, which seem to offer effective control or prevention of cervical cancer, metastatic CCa is an incurable tumour for which new anticancer agents and therapeutic strategies are urgently needed (3, 4).

Nanoparticles (NPs) are new materials with dimension between approximately 1 and 100 nm in at least one dimension and in as many as three dimensions (5). Due to their physiochemical properties and characteristics, NPs exhibit therapeutic potential for numerous diseases (6). Silver nanoparticles (AgNPs) constitute a new type of nanometal particle that has been widely used in biological, medical, and engineering science (6). In particular, synthetic biologically active AgNPs have exhibited extensive therapeutic potential with broad spectrum antibacterial, antifungal, anticancer, anti-inflammatory, wound healing, antioxidative, and anti-diabetic activity (7–9).

In this study, our group applied an aqueous *Ginkgo biloba* leaf extract (GB-extract) as a reducing agent during AgNPs synthesis. *G. biloba*, commonly known as gingko or ginkgo, is classified in the Ginkgoaceae family and Ginkgo genus (10). Gingko leaf extracts have obvious effects as treatments for coronary heart disease, angina pectoris and hyperlipidaemia, and they exhibit anticancer, anti-inflammatory, antioxidant, anti-allergic, anti-ulcerogenic, and antibacterial activities because they are enriched in active metabolites, such as vitamin C, vitamin E, octacosanol, β-sitosterol, stigmasterol, alkaloids, and a variety of flavonoids (11, 12). In our study, we found that gingko leaf extract-based synthesis of silver nanoparticles (GB-AgNPs) exhibited good inhibitory effects on cell proliferation and induced the apoptosis of CCa cells. However, the molecular mechanism by which GB-AgNPs affect on CCa cells remains unknown.

Several studies have shown correlations between the activation of apoptosis and various synthetic AgNPs based on different medicinal plants. Recent evidence indicates that different mechanisms are involved in AgNP-induced apoptosis; such as ER stress mediation, altered ubiquitination, changed intracellular calcium (Ca^2+^) concentration, and induced reactive oxygen species (ROS) production (13–16). In this study, we analyzed the characteristics of GB-AgNPs and the inhibition of cervical cancer cell proliferation to investigate the underlying mechanism of GB-AgNP-induced apoptosis in cervical cancer cell lines.

## Materials and Methods

### Chemicals, Solvents, and Antibodies

For cell cultures, DMEM, FBS, penicillin, streptomycin, and trypsin-EDTA were purchased from GE Life (HyClone, USA). DMSO, MTT, MTS, and Hoechst 33258 were purchased from Sigma (Santa Clara, USA). An annexin V-FITC/PI apoptosis detection Kit, reactive oxygen species (ROS) assay kit, malondialdehyde (MDA) assay kit, glutathione peroxidase (GSH-Px) assay kit and superoxide dismutase (SOD) assay kit were purchased from KeyGen Biotech (Beijing, CN).The primary antibodies against β-actin, Bax, Bcl-2, Caspase-9, Caspase-3, Cyt C, and Vadc 1 were purchased from Cell Signaling (Massachusetts, USA). Horseradish peroxidase-conjugated goat anti-rabbit antibody was also purchased from Cell Signaling and was used as the secondary antibody. All the other fine chemicals and solvents used for this study were of analytical grade.

### The Extract of *Ginkgo Biloba* Leaves

*G. biloba* grown in the Taizhou region and ginkgo leaves were purchased from in the local public market (Taizhou, CN). The ginkgo leaves were washed three times with deionized water, freeze-dried, and pulverized through a 40-mesh sieve, and stored in a darkened room at 4 °C. The resulting gingko leaf powder (1.0 g) was dissolved in ultrapure water (60 ml), placed in an ultrasonic bath at 80 °C for 30 min, and cooled. After centrifugation at 10,000 g for 20 min, the supernatant was obtained to generate aqueous extracts of the *G. Biloba* leaves (GB-extracts).

### Synthesis and Characterization of the GB-AgNPs

Different concentrations of GB-extracts and AgNO_3_ were combined to synthesize GB-AgNPs at different temperatures and time. Detailed synthetic conditions are shown in **Supplementary Materials Table 1**. The size of the AgNPs was determined using a nanoparticle size analyser, and the synthesis conditions were determined, with conditions for synthesizing the smallest mean particles found to be the best. The synthesized silver nanoparticle gel solution was freeze-dried to obtain GB-AgNP powder.

The physiochemical properties and characteristics of the GB-AgNPs were analyzed by ultraviolet-visible spectroscopy with absorbance between 200 and 800 nm (Shimadzu, Japan), and the particle size and shape were measured by a NanoBrook Omni nanometre particle size analyser (Brookhaven, USA) and an HT-7700 transmission electron microscope (Hitachi, Japan).

### Cell Lines and Culture

Human cervical epithelial cells (HcerEpic cell), HeLa, and SiHa human CCa cell lines were purchased from ATCC (Manassas, USA). The cells were cultured in DMEM supplemented with FBS (10%, v/v), penicillin (100 U/ml), and streptomycin (100 μg/ml) at 37 °C in a humidified incubator with 5% CO_2_. The cells were passaged when the confluence reached 80%. The cells in the logarithmic growth phase were used for assays.

### Cell Viability and Proliferation Assay

Cell viability and proliferation were determined using MTT, MTS, and colony formation assays were performed as described in our previous reports (17). Briefly, for MTT assay, cells were incubated in 96-well plates and exposed to the indicated concentration of 40, 60, 90 nm GB-AgNPs or GB-extracts or AgNO_3_ or no-treatment control for 24 h. 10 µl MTT (5 mg/ml) was added to each well and incubated for 4 h. The medium was replaced with 150 µl of DMSO to dissolve the crystal formazan dye and absorbance was detected at 540 nm using an ELX808IU Microplate Reader (BioTek, USA). For MTS assay, cells were plated in 96-well plates and exposed to the indicated concentrations of 40 nm GB-AgNPs or no-treatment control for 12, 24, and 36 h. 20 µl of MTS was added to each well and incubated for 1 h, after which the absorbance of the MTS signal was calculated after absorbance detection at 490 nm. For colony formation assay, cells were treated with the indicated amount of 40 nm GB-AgNPs or no-treatment control for 24 h and cultured at a density of 500 cells/well in 6-well plates. Then the medium was changed every 3 d, and after two weeks cell colonies were stained with Giemsa stain solution (Solarbio, CN). Visible colonies were photographed and counted using a Gel DocTMXR^+^ Molecular Imager system (BioRad, USA).

### Hoechst 33258 Staining

Hoechst 33258 staining was performed as described in our previous report (17). Briefly, cells were seeded in 6-well plates and added to indicated concentrations of 40 nm GB-AgNPs or a no-treatment control for 24 h, and then were stained using a Hoechst 33258 Staining Kit according to the manufacturer’s instructions. The morphology of the apoptotic cells was observed, and the number of cells was counted under an IX73-AF12/PH fluorescence microscope (Olympus, Japan).

### Flow Cytometry

Cells were seeded in 6-well plates and added to different concentrations of 40 nm GB-AgNPs or a control for 24 h. The apoptosis rate was measured by an Annexin-V FITC/PI staining kit according to the manufacturer’s protocol. These data were analyzed using FlowJo software (BD Biosciences, USA).

### ROS and Oxidative Stress Measurements

The intracellular ROS level and oxidative stressindexes were measured by a ROS assay kit, MDA assay kit, GSH-Px assay kit, and SOD assay kit according to the manufacturers’ instructions.

### Western Blotting

Western blotting was performed as described in our previous reports (17). Briefly, cells were homogenized in RIPA lysis buffer. Total protein extracts and concentrations were measured using the BCA protein assay kit (Pierce, 23225). Proteins were separated on 12% SDS-PAGE and transferred to PVDF (Bio-Rad, USA) membranes. After blocking, the membranes were incubated with primary antibodies at 4°C overnight, and were subsequently incubated with secondary antibodies at room temperature for 1 h. Target proteins were detected and quantified using Enhanced Chemiluminescence reagents (Millipore, WBKLS0100).

### Statistical Analysis

Statistical analyses were performed with Prism 7 software. Differences were analyzed by one-way ANOVA or two-sample equal variance Student’s *t* test. Data are expressed as the means ± SD. A value of *P* < 0.05 was considered significant.

## Results

### Characterization of the Synthesized GB-AgNPs

**Figure 1:**
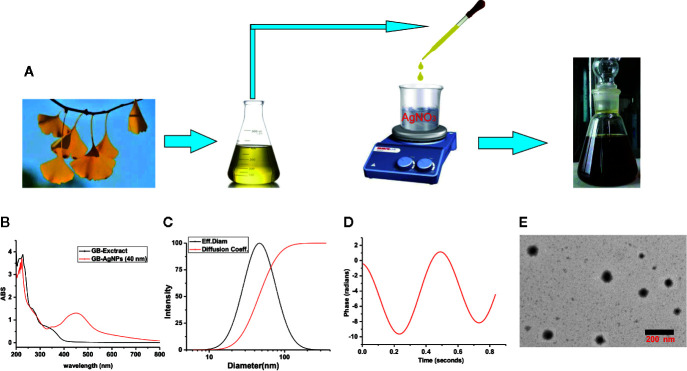
Schematic representation of silver nanoparticle synthesis with aqueous extracts of *G.biloba* leaves. **(A)** Step silver nanoparticle (AgNP) synthesis; **(B)** UVspectrum; **(C)** dynamic lights scattering (DLS) images; **(D)** Zeta-potential images; and **(E)** TEM images.

During GB-AgNPs synthesis, the colour of the reaction solution changed significantly, and eventually, the colourless solution gradually became brown-orange, suggesting the surface plasmon resonance excitation of the synthesized AgNPs (**Figure 1A**). As shown in **Figure 1B**, the UV-Vis spectra were recorded for the GB*-*extracts and synthetic GB-AgNPs, with the optical characteristic peak of nanoparticles at approximately 400–450 nm. The absorption spectrum of the GB-AgNPs spanned a wide range, from 320 to 600 nm, with a prominent peak at 448 nm, while the GB-extract shown no similar peak between 400 and 600 nm.

To assess the size, the distribution of the synthesized AgNPs with the GB-extract, the dynamic lights scattering (DLS) method was used. In the series of silver nanoparticles we synthesized (**Supplementary Table 1** and **Figure 1**), the smallest average particle size was 40.2 ± 1.2 nm with low polydispersity (0.091 ± 0.011), and the zeta potential values was measured -34.56 mV with low mobility (-2.95), meantime no particle agglomeration was observed (**Figures 1C, D**). Moreover, to analyze the size and physical characteristics of the GB-AgNPs, transmission electron microscopy was performed. As shown in **Figure 1E**, the shape of the GB-AgNPs seemed spherical or oval with diameters between 90 and 20 nm on average. These results supported and statistically correlated with the DLS images of the GB-AgNPs. In addition, the UV absorption spectrum and particle size of synthesized GB-AgNPs (40 nm) were monitored in two weeks, and there was no significant change to be observed, which shows that our synthesized GB-AgNPs have good stability (**Supplementary Figures 2 and 3**). These results suggest that we could use aqueous extracts of the *G. Biloba* leaves as a reducing agent to prepare a series of stabilized AgNPs.

### Cytotoxic Effects of the GB-AgNPs on CCa Cells

**Figure 2:**
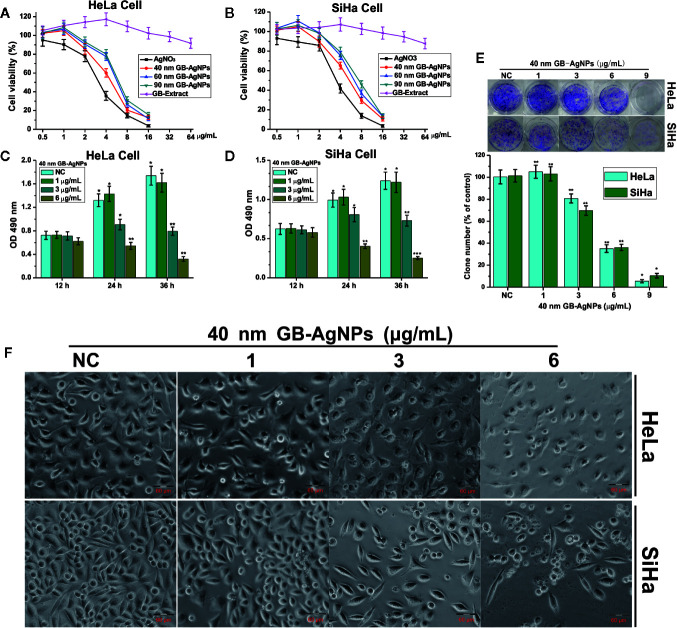
Ginkgo extract synthesized silver nanoparticles (GB-AgNPs) inhibit the proliferation of cervical cancer cells. **(A, B)** HeLa and SiHa cells were treated with different sizes of GB-AgNPs or AgNO_3_ or GB-Extract for 24 h, and cell viability was measured with an MTT assay; **(C, D)** HeLa and SiHa Cells were treated with indicated concentrations of 40 nm GB-AgNPs for 12, 24, and 36 h, and MTS-incorporating live cells were detected with an MTS proliferation assay; **(E)** Cells were treated with indicated concentrations of 40 nm GB-AgNPs for 14 days, and live cells were detected with a colony formation assay; **(F)** cells were treated with indicated concentrations of 40 nm GB-AgNPs for 24 h, cell morphology was observed under an inverted phase-contrast microscope and images were obtained. Data are expressed as the means ± SD; *P < 0.05, **P < 0.01, ***P < 0.001.

To assess the cytotoxic effects of green-synthesized GB-AgNPs, the cell growth and proliferation of two CCa lines, HaLa and SiHa, were determined. First, these two cell lines were exposed to different concentrations and sizes of GB-AgNPs or different concentrations of GB-Extract and AgNO_3_ for the indicated durations, followed by analysis of whether the GB-AgNPs affected cell viability by MTT assay. The results of the MTT assays suggested that the viability of both the HaLa and SiHa cells was not only dose-dependent but also size-dependent with the viability suppression by GB-AgNPs greater than that by the controls (**Figures 2A, B**). In addition, the various sizes of the GB-AgNPs not only failed to inhibit cell growth but also promoted cell growth at low concentrations: cell growth was inhibited only when the concentration was greater than 1.5 mg/ml. At the same time, the cytotoxicity of AgNO_3_ was greater than that of all GB-AgNPs at the same concentration, while the GB extract was relatively less cytotoxic at high concentrations (**Figures 2A, B**).

We also assessed whether PAC is toxic to normal human gastric mucosal cells (GES-1), and the results showed that PAC at <1.6 µM was not cytotoxic to normal gastric cells; the dose that produced obvious cytotoxicity was ∼3.2 µM, more than eightfold the dose used in our assays.

Subsequently, to examine whether the 40 nm GB-AgNPs affected cell proliferation, MTS and colony formation assays were performed. The MTS assays showed that cytotoxic effects of the 40 nm GB-AgNPs on both the HaLa and SiHa cells not only increased with exposure time but also with increasing doses (**Figures 2C, D**). Similarly, the colony formation assays indicated that 40 nm GB-AgNPs treatment markedly inhibited that proliferation of these two CCa cell lines compared to the controls (**Figure 1E**). Moreover, microscopy images showed that, compared with the controls, both the HaLa and SiHa cells had noticeably increased cellular atrophy and decreased cellular attachment after 24 h of exposure to the 40 nm GB-AgNPs (**Figure 2F**). Additionally, the toxicity of 40 nm GB-AgNPs to normal human cervical epithelial cells (HcerEpic) was assessed and the results show that the dose of 40 nm GB-AgNPs (>8.0 µg/ml) has obvious cytotoxicity to normal cervical epithelial cells (**Supplementary Figure 4**). All the results indicate that GB-AgNPs could specifically suppress the proliferation and viability of the cervical cells. As shown in **Figures 2A, B**, the IC_50_ value of 40 nm GB-AgNPs was approximately 3 μg/ml for both HeLa and SiHa cells.

Furthermore, colony formation assays suggested that PAC treatment markedly suppressed proliferation in MGC-803 and SGC-7901 cells compared to controls (**Figure 1E**). We also assessed whether PAC is toxic to normal human gastric mucosal cells (GES-1), and the results showed that PAC at <1.6 µM was not cytotoxic to normal gastric cells; the dose that produced obvious cytotoxicity was ∼3.2 µM, more than eightfold the dose used in our assays (**Supplementary Figure 1**).

### GB-AgNPs Induced the Apoptosis of the CCa Cells

**Figure 3:**
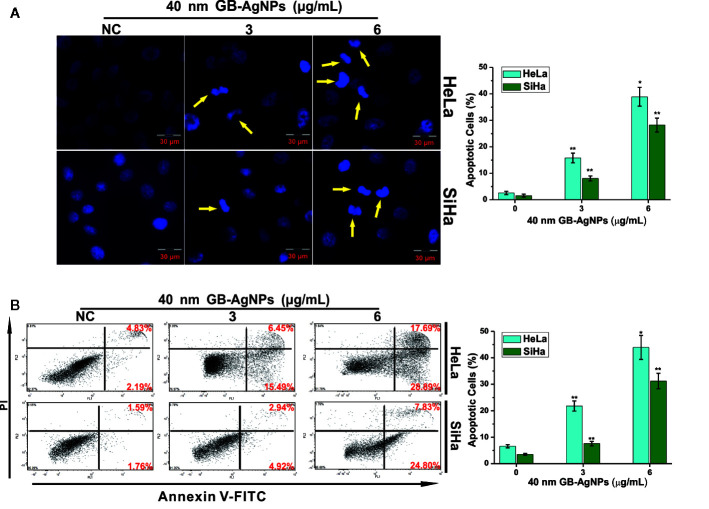
Ginkgo extract synthesized silver nanoparticles (GB-AgNPs) induce apoptosis in cervical cancer cells. **(A)** HeLa and SiHa cells were treated with the indicated concentrations of 40 nm GB-AgNPs for 24 h and stained with Hoechst 33258. Typical apoptotic morphology was observed in treated cells compared to untreated cells; **(B)** Cells were treated as in **(A)** for 24 h, apoptotic cells were stained by PI/annexin-V, and flow cytometry was performed. Data are expressed as the means ± SD; *P < 0.05, **P < 0.01.

Next, we examined whether the suppression of cell proliferation after GB-AgNP treatment was accompanied by apoptosis. HaLa and SiHa cells were exposed to different amounts of 40 nm GB-AgNPs for 24 h, followed by Hoechst 33258 staining to examine whether apoptosis occurs. Hoechst 33258 staining showed changes in nuclear morphology, including cell nucleus shrinkage and chromatin condensation, which are typical apoptotic morphological features, after the 40 nm GB-AgNPs (6 μg/ml) were added to these two CCa cell lines (**Figure 3A**). Furthermore, to verify the apoptotic effects of the GB-AgNPs on HaLa and SiHa cells, we carried out flow cytometry on Annexin V-FITC/PI stained cells and discovered that GB-AgNPs induced both early and late apoptosis in a dose-dependent manner. **Figure 3B** clearly shows that the percent of apoptotic HeLa cells was 6.57 ± 0.62% for the control group and was increased to 21.78 ± 1.91%, and 43.92 ± 4.53% for the treatment groups; for the SiHa cells, the percent of apoptotic cells was 3.51 ± 3.51% for the control group and was 7.57 ± 0.85% and 31.23 ± 2.93% for the treatment groups. These findings confirmed that GB-AgNPs can induce the apoptosis of CCa cells in a dose-dependent manner.

### GB-AgNP Induced Oxidative Stress and ROS Generation

**Figure 4:**
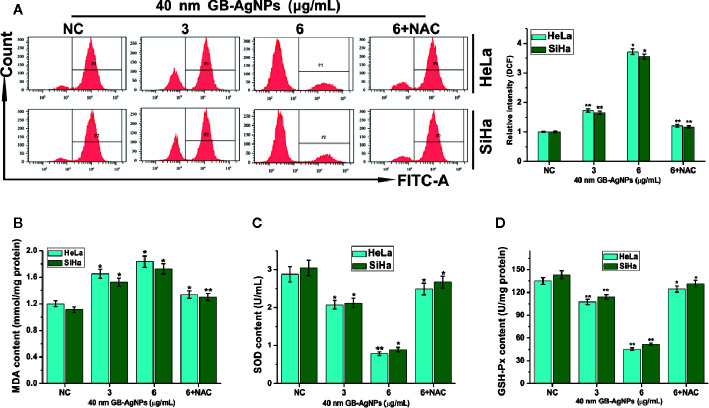
Ginkgo extract synthesized silver nanoparticle (GB-AgNP)-induced oxidative stress in cervical cancer cells. **(A)** HeLa and SiHa cells were treated with various doses of 40 nm GB-AgNPs for 24 h, and then intracellular ROS were measured by flow cytometry; **(B)** the content of lipid peroxidation MDA in cells was measured; **(C)** the level of SOD was measured in the cells; and **(D)** the level of GSH-Px was measured in the cells. Data are expressed as the means ± SD; *P < 0.05, **P < 0.01.

AgNPs can effectively block the respiratory chain of bacteria and thus have antibacterial effects (18). The production of intracellular ROS plays a critical role in oxidative stress and apoptosiss (19). Therefore, we assessed the oxidative stress and ROS levels, which play important role in the apoptosis induced by various anticancer agents, in both CCa cell lines after GB-AgNPs treatment. As shown in **Figure 4**, upon 40 nm GB-AgNPs treatment, the levels of intracellular ROS and malondialdehyde (MDA), an endproduct of lipid oxidation, were sharply increased compared with those of the control group (**Figures 4A, B**), whereas the levels of antioxidant enzymes such as GSH-Px and SOD were remarkably decreased (**Figures 4C, D**). Additionally, when the 40 nm GB-AgNPs (6 μg/ml) with 20 μM N-acetyl-L-cysteine (NAC, an ROS scavenger) were added, the levels of ROS and MDA, which had sharply increased, were significantly reduced, and the originally low content levels of SOD and GSH-Px were obviously increased. These results showed that GB-AgNPs can induce oxidative stress by generating intracellular ROS.

### GB-AgNPs Induced Apoptosis *Via* the Mitochondrial Pathway

**Figure 5:**
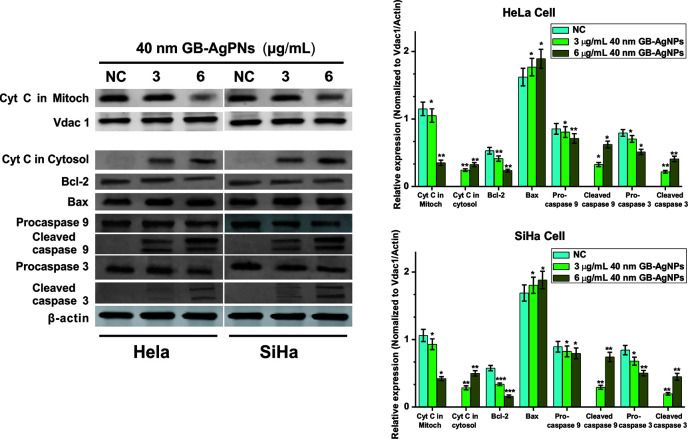
Ginkgo extract synthesized silver nanoparticles (GB-AgNPs) induce apoptosis through caspase-dependent mitochondrial signalling pathways. Expression levels of apoptosis-related proteins were detected by western blot analysis after HeLa and SiHa cervical cancer cells were treated with the indicated concentrations of 40 nm GB-AgNPs. Data are expressed as the means ± SD; *P < 0.05, **P < 0.01.

The overproduction of ROS may cause mitochondrial dysfunction or damage, which may affect the expression of some antiapoptotic and proapoptotic genes in the mitochondria (19). We then analyzed the expression levels of antiapoptotic Bcl-2 protein and proapoptotic Bax protein by western blotting. As shown in **Figure 5**, upon 40 nm GB-AgNP treatment, the expression levels of Bcl-2 were decreased in the HeLa and SiHa cells, while the expression levels of Bax were obviously increased in both CCa cell lines. Considering that Bax activation would change the permeabilization of the outer mitochondrial membrane and cause about the release of some proapoptotic mitochondrial proteins into cytosol, the release of cytochrome c (Cyt C) was analyzed (20). Compared with the control group, the content level of Cyt C in the mitochondria was sharply decreased in a dose-dependent manner in these two CCa cell lines after 24 h of exposure to GB-AgNPs; in contrast, the expression of Cyt C in the cytosol was markedly increased by GB-AgNPs treatment of the HeLa and SiHa cells (**Figure 5**). Cyt C released into the cytoplasm can induce apoptosis by activating downstream caspase-dependent apoptotic proteins; therefore, the expression of caspase-9 and caspase-3 was measured. As shown in **Figure 5**, the expressions levels of cleaved caspase-9 and caspase-3 were obviously increased in a dose-dependent manner in the HeLa and SiHa cells treated with GB-AgNPs; whereas the expression levels of pro-caspase 9 and pro-caspase 3 were noticeably decreased in both CCa cell lines. These results clearly indicate that GB-AgNPs induced the apoptosis of cervical cancer cells by generating excess ROS that permeabilized of the outer mitochondrial membrane and increase the amount of Cyt C released from the mitochondria into the cytosol.

**Figure 6:**
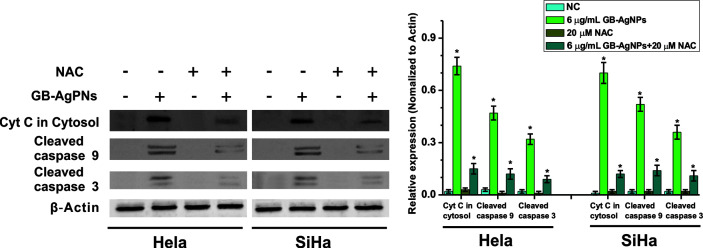
Ginkgo extract synthesized silver nanoparticles (GB-AgNPs) induce apoptosis through mediated intracellular ROS generation. HeLa and SiHa cells were treated with or without NAC (20 μM) for 4 h and then cells were incubated with GB-AgNPs (6 μg/ml) for 24 h, and the expression levels of apoptosis-related proteins were measured by western blot analysis. Data are expressed as the means ± SD; *P < 0.05.

Furthermore, to confirm that GB-AgNPs induced apoptosis through the ROS/Cyt C signaling pathway, we employed the ROS scavenger NAC to measure the effects of Cyt C in the cytosol and caspase-9 and caspase-3 on the apoptosis induced by GB-AgNPs. As shown in **Figure 6**, the effects of the GB-AgNPs on Cyt C release in the cytosol and the cleavage of caspase-9 and caspase-3 were diminished by 20 μM NAC treatment. This finding indicated that NAC blocked the apoptosis induced by the GB-AgNPs, which corroborates the ROS assay results describe above.

## Discussion

In recent decades, AgNPs, particularly green-biosynthesized AgNPs, have exhibited promising potency for biomedical applications due to their powerful antimicrobial properties, effective inhibition of tumour cell proliferation, potent anti-inflammatory and wound healing effects, and substantial chemical stability and biocompatibility (9, 21, 22). On the one hand, green biosynthesis of AgNPs can be undertaken using different natural resources, such as microorganisms, fruit extracts, and medicinal plants, to minimize the amount of stabilizing agents, which adsorb different biologically active substances during synthesis, endowing AgNPs with different biological activities (23). On the other hand, green-synthesized AgNPs are generally less toxic to mammalian cells and more environmental friendly than AgNPs synthesized by other methods (24). Biosynthetic AgNPs are larger than the chemosynthetic and physic synthetic AgNPs (9). Interestingly, we know that smaller AgNPs induces greater cytotoxicity, and we verified this phenomenon by MTT assay. Herein, a series of GB-AgNPs were prepared using different concentrations of gingko aqueous extracts, and nanoparticles smaller than 100 nm had narrow distribution and a good coefficient of dispersion, which means that the GB-AgNPs can easily pass through the vascular gap in capillary tumour tissue (25).

The commonly known notable hallmark of cancer is uncontrolled proliferation, which may indicate dysfunctional apoptotic machinery in the cell (4). We first tested whether the GB-AgNPs are capable of inhibiting the proliferation of two CCa cell lines. The results of the MTT, MTS and colony formation assays showed that the GB-AgNPs obviously inhibited the growth and proliferation of HeLa and SiHa cells in a dose-, size-, and time-dependent manner. Second, we discovered that GB-AgNPs induced apoptosis by generating ROS and increasing oxidative stress, which is usually involved in cell apoptosis and mitochondrial dysfunction (26). As expected, the levels of intracellular ROS and lipid oxidation end-product MDA were significantly increased after treatment with GB-AgNPs, compared to those of the controls, which affected the mitochondrial respiratory chain complex and key enzyme activity in mitochondria *in vitro* (26). Then, the antioxidant enzymes SOD and GSH-Px were found to be consumed at high levels to abrogate the increased ROS levels. Subsequently, we assessed several relative mitochondrial antiapoptotic and proapoptotic proteins by western blot assays. The Cyt C level in the mitochondria was sharply decreased upon GB-AgNP treatment compared to that of the controls, while the Cyt C level in the cytosol was dramatically increased after treatment with GB-AgNPs compared to the level in the controls, and this increase induced the caspase-dependent apoptosis pathway. Additionally, two CCa cells were treated with GB-AgNPs and the antioxidant NAC, and the increased levels of ROS and Cyt C in the cytosol and the extent of caspase-9 and caspase-3 cleavage were diminished. Our study findings strongly indicate that GB-AgNPs generate excess of ROS and induce apoptosis.

ROS play important roles in the progression of cancers (26). Compared to normal cells, cancer cells usually have higher levels of ROS and oxidative stress (27). However, the effect of ROS on the development of cancer is complex. Several studies have shown that ROS can induce DNA mutation and prooncogenic expression, thus promoting cancer formation (26, 27). ROS can also influence cell survival or proliferation by cellular processes (27, 28). Hence, a powerful strategy to fight cancer may be realized by destroying the balanced of intracellular ROS to induce their accumulation. Many studies have shown that many agents could induce cell apoptosis by generating ROS in a verity of cancer cells (29–31). Interestingly, in different studies, AgNPs were found to be antioxidants or prooxidants promoters (7, 8). These contradictory actions may be due to the synthesis of AgNPs using various medicinal plants, not the size of AgNPs nor the dose or duration of the treatment (8). In this study, high ROS levels were observed in both CCa cell lines treated with the GB-AgNPs, which implies that mitochondrial injury is probably involved in the apoptosis induced by GB-AgNPs. Mitochondria are the main production sites of ROS, and high levels of ROS induces mitochondrial membrane permeability, which lead to the release of several mitochondrial proteins into the cytosol (29). Cyt C is known as a proapoptotic protein and activates downstream caspase-dependent apoptosis when it is released from mitochondria into the cytosol (19). Bcl-2 is an antiapoptotic protein that can maintain the integrity of the mitochondrial membrane and prevent the release of Cyt C into the cytosol (32).

**Figure 7:**
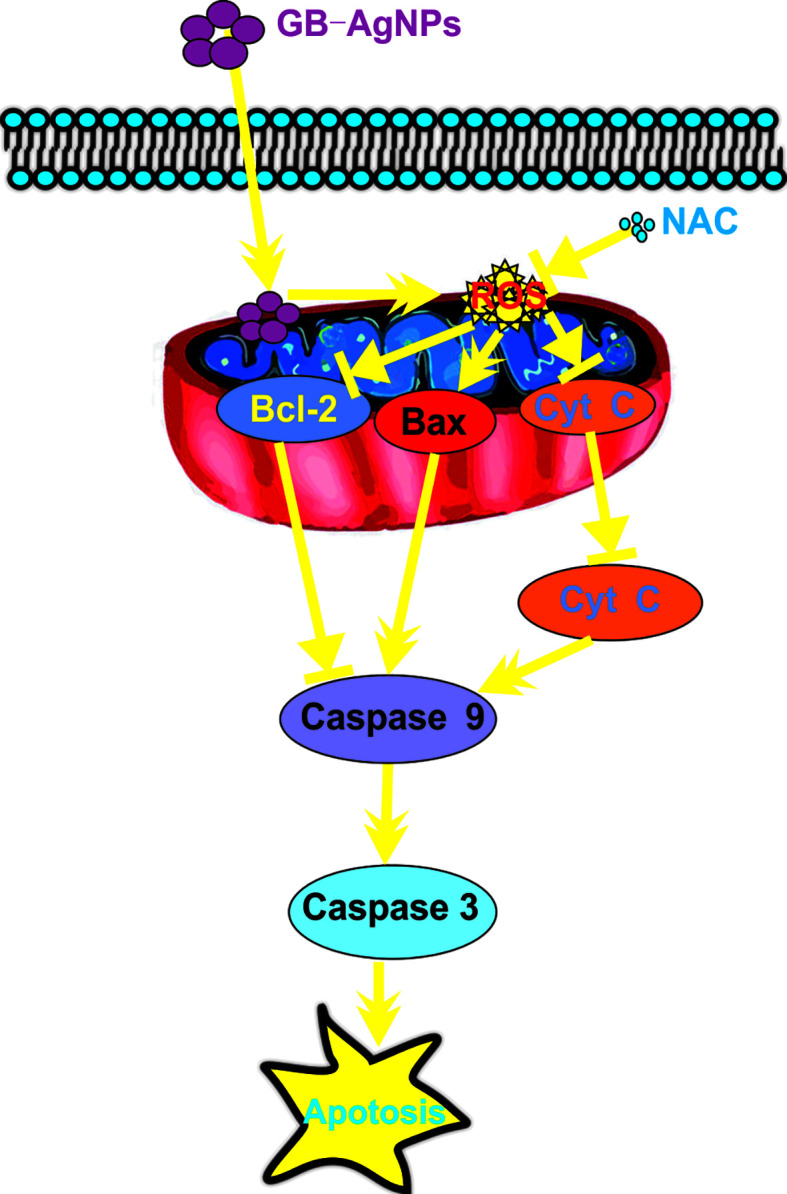
Schematic mechanisms of Ginkgo extract synthesized silver nanoparticle (GB-AgNP)-induced apoptosis by caspase-dependent mitochondrial signalling pathways in cervical cancer cells.

In summary, novel green biosynthetic AgNPs were prepared with aqueous extracts of *G. biloba* leaves, and the molecular mechanism of the effect of GB-AgNPs on human CCa (HeLa and SiHa) cell cytotoxicity and apoptosis was explored. Exposure to GB-AgNPs for 24 h induced an increase in ROS generation and mitochondrial membrane permeability, Cyt C release into the cytosol, and the cleavage of caspase-9 and caspase-3. All assays results indicated that GB-AgNPs contributed to the cell apoptosis of HeLa and SiHa cells (**Figure 7**). Herein, the results of this study offer further evidence of the cytotoxicity and the anticancer efficacy caused by increased ROS levels that induce the caspase-dependent mitochondrial apoptotic signaling pathways, which justifies further exploration of GB-AgNP potential for cancer therapeutics and preventive CCa treatments.

## Data Availability Statement 

All datasets generated for this study are included in the article/**Supplementary Material**.

## Author Contributions

ZX and MW: Data analysis and interpretation. QF: Data analysis writing. HZ: Data collection and analysis. YL: Data collection. SZ: Conception and design, data analysis and interpretation, and writing. All authors contributed to the article and approved the submitted version.

## Funding

This work was supported by the Key Research and Development Project of Hainan Province (Grant No.ZDYF2019177), the Open Fund Project of Hainan Provincial Key Laboratory of Basic Medicine (Grant No.JCKF2020010), and the National Natural Science Foundation of China (Grant No. 81560484).

## Conflict of Interest

The authors declare that the research was conducted in the absence of any commercial or financial relationships that could be construed as a potential conflict of interest.

## References

[1] Siegel RL Miller KD Jemal A Cancer statistics, 2020. CA Cancer J Clin (2020) 70(1):7–30. 10.3322/caac.21590 31912902

[2] Canfell K Kim JJ Brisson M Keane A Simms KT Caruana M Mortality impact of achieving WHO cervical cancer elimination targets: a comparative modelling analysis in 78 low-income and lower-middle-income countries. Lancet (2020) 395(10224):591–603. 10.1016/S0140-6736(20)30157-4 32007142 PMC7043006

[3] Brisson M Kim JJ Canfell K Drolet M Gingras G Burger EA Impact of HPV vaccination and cervical screening on cervical cancer elimination: a comparative modelling analysis in 78 low-income and lower-middle-income countries. Lancet (2020) 395(10224):575–90. 10.1016/S0140-6736(20)30068-4 PMC7043009 32007141

[4] Cohen AC Roane BM Leath CA,3 Novel Therapeutics for Recurrent Cervical Cancer: Moving Towards Personalized Therapy. Drugs (2020) 80(3):217–27. 10.1007/s40265-019-01249-z PMC7033025 31939072

[5] Merz SN Farrell ZJ Dunn CJ Swanson RJ Egorov SA Green DL Theoretical and Experimental Investigation of Microphase Separation in Mixed Thiol Monolayers on Silver Nanoparticles. ACS Nano (2016) 10(11):9871–8. 10.1021/acsnano.6b02091 27744676

[6] Krasniewska K Galus S Gniewosz M Biopolymers-Based Materials Containing Silver Nanoparticles as Active Packaging for Food Applications-A Review. Int J Mol Sci (2020) 21(3):698. 10.3390/ijms21030698 PMC7037217 31973105

[7] Docea AO Calina D Buga AM Zlatian O Paoliello MMB Mogosanu GD The Effect of Silver Nanoparticles on Antioxidant/Pro-Oxidant Balance in a Murine Model. Int J Mol Sci (2020) 21(4):1233. 10.3390/ijms21041233 PMC7072874 32059471

[8] Chinnasamy G Chandrasekharan S Bhatnagar S Biosynthesis of Silver Nanoparticles from Melia azedarach: Enhancement of Antibacterial, Wound Healing, Antidiabetic and Antioxidant Activities. Int J Nanomed (2019) 14:9823–36. 10.2147/IJN.S231340 PMC6913292 31849471

[9] Guilger-Casagrande M de Lima R Synthesis of Silver Nanoparticles Mediated by Fungi: A Review. Front Bioeng Biotechnol (2019) 7:287. 10.3389/fbioe.2019.00287 31696113 PMC6818604

[10] Bader BM Jugelt K Schultz L Schroeder OH Ginkgo biloba L (Ginkgoaceae) Leaf Extract Medications From Different Providers Exhibit Differential Functional Effects on Mouse Frontal Cortex Neuronal Networks. Front Pharmacol (2018) 9:848. 10.3389/fphar.2018.00848 30123130 PMC6085676

[11] Ude C Schubert-Zsilavecz M Wurglics M Ginkgo biloba extracts: a review of the pharmacokinetics of the active ingredients. Clin Pharmacokinet (2013) 52(9):727–49. 10.1007/s40262-013-0074-5 23703577

[12] Omidkhoda SF Razavi BM Hosseinzadeh H Protective effects of Ginkgo biloba L. against natural toxins, chemical toxicities, and radiation: A comprehensive review. Phytother Res (2019) 33(11):2821–40. 10.1002/ptr.6469 31429152

[13] Zhang K Liu X Samuel Ravi SOA Ramachandran A Aziz Ibrahim IA Nassir AM Synthesis of silver nanoparticles (AgNPs) from leaf extract of Salvia miltiorrhiza and its anticancer potential in human prostate cancer LNCaP cell lines. Artif Cells Nanomed Biotechnol (2019) 47(1):2846–54. 10.1080/21691401.2019.1638792 31299869

[14] Prateeksha BR S VK G Deeba F Bajpai R Pandey V Non-Toxic and Ultra-Small Biosilver Nanoclusters Trigger Apoptotic Cell Death in Fluconazole-Resistant Candida albicans via Ras Signaling. Biomolecules (2019) 9(2):47. 10.3390/biom9020047 PMC6406502 30769763

[15] Li L Li L Zhou X Yu Y Li Z Zuo D Silver nanoparticles induce protective autophagy via Ca(2+)/CaMKKbeta/AMPK/mTOR pathway in SH-SY5Y cells and rat brains. Nanotoxicology (2019) 13(3):369–91. 10.1080/17435390.2018.1550226 30729847

[16] Kanipandian N Li D Kannan S Induction of intrinsic apoptotic signaling pathway in A549 lung cancer cells using silver nanoparticles from Gossypium hirsutum and evaluation of in vivo toxicity. Biotechnol Rep (Amst) (2019) 23:e00339. 10.1016/j.btre.2019.e00339 31467862 PMC6713847

[17] Wang M Zhao H Hu J Xu Z Lin Y Zhou S A New Azaphilone, Induces Apoptosis in Gastric Cancer by Blocking the Notch Signaling Pathway. Front Oncol (2020) 10:116. 10.3389/fonc.2020.00116 32117763 PMC7026506

[18] Sanyasi S Majhi RK Kumar S Mishra M Ghosh A Suar M Polysaccharide-capped silver Nanoparticles inhibit biofilm formation and eliminate multi-drug-resistant bacteria by disrupting bacterial cytoskeleton with reduced cytotoxicity towards mammalian cells. Sci Rep (2016) 6:24929. 10.1038/srep24929 27125749 PMC4850392

[19] You L Yang C Du Y Liu Y Chen G Sai N Matrine Exerts Hepatotoxic Effects via the ROS-Dependent Mitochondrial Apoptosis Pathway and Inhibition of Nrf2-Mediated Antioxidant Response. Oxid Med Cell Longev (2019) 2019:1045345. 10.1155/2019/1045345 31737162 PMC6815593

[20] Yao W Lin Z Wang G Li S Chen B Sui Y Delicaflavone induces apoptosis via mitochondrial pathway accompanying G2/M cycle arrest and inhibition of MAPK signaling cascades in cervical cancer HeLa cells. Phytomedicine (2019) 62:152973. 10.1016/j.phymed.2019.152973 31177019

[21] Gnanakani PE Santhanam P Premkumar K Eswar Kumar K Dhanaraju MD Nannochloropsis Extract-Mediated Synthesis of Biogenic Silver Nanoparticles, Characterization and In Vitro Assessment of Antimicrobial, Antioxidant and Cytotoxic Activities. Asian Pac J Cancer Prev (2019) 20(8):2353–64. 10.31557/APJCP.2019.20.8.2353 PMC6852812 31450906

[22] Villeret B Dieu A Straube M Solhonne B Miklavc P Hamadi S Silver Nanoparticles Impair Retinoic Acid-Inducible Gene I-Mediated Mitochondrial Antiviral Immunity by Blocking the Autophagic Flux in Lung Epithelial Cells. ACS Nano (2018) 12(2):1188–202. 10.1021/acsnano.7b06934 29357226

[23] Sathiyaseelan A Saravanakumar K Mariadoss AVA Wang MH Biocompatible fungal chitosan encapsulated phytogenic silver nanoparticles enhanced antidiabetic, antioxidant and antibacterial activity. Int J Biol Macromol (2020) 153:63–71. 10.1016/j.ijbiomac.2020.02.291 32112842

[24] Pedziwiatr-Werbicka E Horodecka K Shcharbin D Bryszewska M Nanoparticles in combating cancer: Opportunities and limitations. A brief review. Curr Med Chem (2020) 27. 10.2174/0929867327666200130101605 32000637

[25] Erdogan O Abbak M Demirbolat GM Birtekocak F Aksel M Pasa S Green synthesis of silver nanoparticles via Cynara scolymus leaf extracts: The characterization, anticancer potential with photodynamic therapy in MCF7 cells. PLoS One (2019) 14(6):e0216496. 10.1371/journal.pone.0216496 31220110 PMC6586393

[26] Shadel GS Horvath TL Mitochondrial ROS signaling in organismal homeostasis. Cell (2015) 163(3):560–9. 10.1016/j.cell.2015.10.001 PMC4634671 26496603

[27] Yamada M Han X Benfey PN RGF1 controls root meristem size through ROS signalling. Nature (2020) 577(7788):85–8. 10.1038/s41586-019-1819-6 PMC6930331 31801996

[28] Lee CH Ying TH Chiou HL Hsieh SC Wen SH Chou RH Alpha-mangostin induces apoptosis through activation of reactive oxygen species and ASK1/p38 signaling pathway in cervical cancer cells. Oncotarget (2017) 8(29):47425–39. 10.18632/oncotarget.17659 PMC5564576 28537893

[29] Yee C Yang W Hekimi S The intrinsic apoptosis pathway mediates the pro-longevity response to mitochondrial ROS in C. elegans. Cell (2014) 157(4):897–909. 10.1016/j.cell.2014.02.055 24813612 PMC4454526

[30] Anupama N Preetha Rani MR Shyni GL Raghu KG Glucotoxicity results in apoptosis in H9c2 cells via alteration in redox homeostasis linked mitochondrial dynamics and polyol pathway and possible reversal with cinnamic acid. Toxicol In Vitro (2018) 53:178–92. 10.1016/j.tiv.2018.08.010 30144576

[31] Fu SC Liu JM Lee KI Tang FC Fang KM Yang CY Cr(VI) induces ROS-mediated mitochondrial-dependent apoptosis in neuronal cells via the activation of Akt/ERK/AMPK signaling pathway. Toxicol In Vitro (2020) 65:104795. 10.1016/j.tiv.2020.104795 32061800

[32] Lv J Guan W You Q Deng L Zhu Y Guo K RIPC provides neuroprotection against ischemic stroke by suppressing apoptosis via the mitochondrial pathway. Sci Rep (2020) 10(1):5361. 10.1038/s41598-020-62336-w 32210331 PMC7093414

