# Supplementary material for: Green Biosynthesized Silver Nanoparticles With Aqueous Extracts of Ginkgo Biloba Induce Apoptosis via Mitochondrial Pathway in Cervical Cancer Cells
Source: Front Oncol. 2020 Oct 20;10:575415. doi: 10.3389/fonc.2020.575415 (PMC7606942; doi:10.3389/fonc.2020.575415)
Supplement: Supplementary file 1 [file Table_1.docx]

Supplementary Material

# Supplementary Tables

**Supplementary Table 1.** The green biosynthesis conditions and results of silver nanoparticles using aqueous extract of *Ginkgo Biloba* (Total volume: 10 mL)

| GB-extract (mg/mL) | AgNO_3_  (mg/mL) | Temperature (̊C) | Rotate speed (rpm) | Time (h) | Average particle size (nm) | Zeta potential values (mV) |
| --- | --- | --- | --- | --- | --- | --- |
| 1.1167 | 0.5084 | 25 | 150 | 6 | 40.2±1.2 | -34.56 |
| 1.1167 | 0.5084 | 20 | 150 | 6 | 102±2.2 | -14.96 |
| 1.1167 | 0.5084 | 30 | 150 | 6 | 68±1.4 | -24.97 |
| 1.1167 | 0.5084 | 25 | 150 | 4 | 130±2.1 | invalid |
| 1.1167 | 0.5084 | 25 | 150 | 8 | 42±1.1 | -33.47 |
| 1.1167 | 0.5084 | 25 | 150 | 12 | 110±2.4 | -12.22 |
| 0.7865 | 0.5084 | 25 | 150 | 6 | 45±1.0 | -32.78 |
| 0.5583 | 0.5084 | 25 | 150 | 6 | 65±1.3 | -25.20 |
| 1.5765 | 0.5084 | 25 | 150 | 6 | 56±1.7 | -30.12 |
| 1.5765 | 1.0168 | 25 | 150 | 6 | 60±1.3 | -28.72 |
| 1.1167 | 1.0168 | 25 | 150 | 6 | 49±1.2 | -32.16 |
| 2.8941 | 1.0168 | 25 | 150 | 6 | 71±1.3 | -24.70 |
| 2.8941 | 0.7626 | 25 | 150 | 6 | 82±1.1 | -19.62 |
| 3.075 | 1.0165 | 25 | 150 | 6 | 89±1.4 | -18.58 |
| 3.075 | 1.2146 | 25 | 150 | 6 | 72±1.3 | -24.57 |
| 3.075 | 1.8218 | 25 | 150 | 6 | 153±2.5 | invalid |

# Supplementary Figures

**Supplementary Figure 1.** DLS images for a series of green biosynthesized GB-AgNPs (40, 49, 60, 71, 82, 89, 102 nm)

**Supplementary Figure 2.** UV spectrum about 40 nm GB-AgNPs in different time.

**Supplementary Figure 3.** DLS image about 40 nm GB-AgNPs in different time.

**Supplementary Figure 4.** HcerEpic cells were treated with different concentrations of 40 nm GB-AgNPs for 24 h, respectively, and the cell growth inhibitory ration was determined by MTT assay. Data are expressed as the means ± SD; *** *P* < 0.001.
